# An invasion front gene expression signature for higher-risk patient selection in stage IIA MSS colon cancer

**DOI:** 10.3389/fonc.2024.1367231

**Published:** 2024-04-19

**Authors:** Eva Budinská, Martina Čarnogurská, Tina Catela Ivković, Táňa Macháčková, Marie Boudná, Lucie Pifková, Ondřej Slabý, Beatrix Bencsiková, Vlad Popovici

**Affiliations:** ^1^ RECETOX, Faculty of Science, Masaryk University, Brno, Czechia; ^2^ Central European Institute of Technology, Masaryk University, Brno, Czechia; ^3^ Department of Biology, Faculty of Medicine, Masaryk University, Brno, Czechia; ^4^ Department of Comprehensive Cancer Care, Masaryk Memorial Cancer Institute, Brno, Czechia

**Keywords:** colon cancer, invasion front, early stage, prognostic signature, stage II/MSS

## Abstract

Stage II colon cancer (CC) encompasses a heterogeneous group of patients with diverse survival experiences: 87% to 58% 5-year relative survival rates for stages IIA and IIC, respectively. While stage IIA patients are usually spared the adjuvant chemotherapy, some of them relapse and may benefit from it; thus, their timely identification is crucial. Current gene expression signatures did not specifically target this group nor did they find their place in clinical practice. Since processes at invasion front have also been linked to tumor progression, we hypothesize that aside from bulk tumor features, focusing on the invasion front may provide additional clues for this stratification. A retrospective matched case-control collection of 39 stage IIA microsatellite-stable (MSS) untreated CCs was analyzed to identify prognostic gene expression-based signatures. The endpoint was defined as relapse within 5 years vs. no relapse for at least 6 years. From the same tumors, three different classifiers (bulk tumor, invasion front, and constrained baseline on bulk tumor) were developed and their performance estimated. The baseline classifier, while the weakest, was validated in two independent data sets. The best performing signature was based on invasion front profiles [area under the receiver operating curve (AUC) = 0.931 (0.815–1.0)] and contained genes associated with KRAS pathway activation, apical junction complex, and heme metabolism. Its combination with bulk tumor classifier further improved the accuracy of the predictions.

## 1 Introduction

Despite important progress made in early detection and treatment over the last decades, colon cancer (CC) is still one of the major causes of death among all solid tumor cancers accounting for more than 600,000 deaths yearly (1). The TNM (tumor–node–metastasis) staging remains the cornerstone of patient management and outcome prediction, even though several other predictors have been proposed, including commercially available gene signatures, such as Oncotype Dx Colon (2), ColoPrint (3), and ColDX (4), or immune system scoring, such as Immunoscore Colon (5). Globally, stage II CC, accounting for 35%–40% of newly diagnosed cases (SEER Cancer Stat Facts: Colorectal Cancer; https://seer.cancer.gov/statfacts/html/colorect.html), has a good prognosis, with 5-year relative survival rates of 58%–87% (6). However, compared to other stages, it is more heterogeneous with low, intermediate, and high risk for metastatic dissemination subgroups, as recognized in the revised categorization (6). Microsatellite instability (MSI) or deficiency in DNA mismatch repair (dMMR) are characteristics of a low-risk group, with more than 90% 5-year overall survival (7). The high-risk (pT4bN0, stage IIC) or intermediate-risk microsatellite-stable (pT4aN0/MSS, stage IIB/MSS) patients are generally treated with adjuvant chemotherapy after curative surgical resection (8). The benefits from adjuvant therapy are not clear in these patients probably because direct evidence from clinical trials is still insufficient (9). However, the low-risk patients (pT3N0, stage IIA) are usually spared the adjuvant treatment, but still, approximately 13% of them will die within 5 years (6). Therefore, it is of utmost importance to develop better prognostic tools, eventually integrated with the TNM staging, targeting the earlier stage where the benefit from adjuvant treatment may potentially be significant.

All the transcriptomic signatures proposed so far considered whole-tumor sampling for RNA extraction. Still, mounting evidence suggests that processes taking place at the invasion front would be equally prognostic, if not even more. The activation of epithelial-to-mesenchymal transition (EMT) at aberrant expression of nuclear β-catenin as invasion front markers of tumor progression has been recognized previously (10, 11). Also, the infiltrative configuration of the invasion front and the presence of tumor budding have been recognized as additional prognostic parameters (12, 13). It has been proposed that the balance of pro- and anti-tumor factors at the invasion front may be decisive for tumor progression (14) and overexpression of *ZEB2* (an epithelial-to-mesenchymal transition-associated gene) as the invasion front has been identified as an independent prognostic factor in a general CC patient population (15). Additionally, the immune reaction scored along the invasion front could be used to stratify the CC patients into three distinct risk groups (5). In addition, the histopathologic characteristics of the reactive stroma at the invasion front have been shown to bear prognostic potential (16). Thus, it is of interest whether transcriptomics of the invasion front may bring novel discriminative markers that could improve patient stratification.

The goal of the present pilot study is twofold: to assess the prognostic utility of invasion front gene expression and develop a predictor of early relapse within the low-risk stage IIA/MSS colon cancers. From the same group of patients, we develop gene signatures from both bulk tumor (traditional tumor sampling) and tumor invasion front predicting the risk of relapse, and we compare their performance. As the study has a limited sample size, we opted for increasing the contrasts between the groups by selecting patients with relapse within 5 years vs. patients with no relapse for at least 6 years.

## 2 Materials and methods

### 2.1 Samples

This retrospective matched case-control study used tumor samples from patients with CC who underwent surgery at Masaryk Memorial Cancer Institute, Brno, Czech Republic, in the years 1998–2018. Inclusion criteria for this study were as follows: age >18 years, clinically and histopathologically confirmed diagnosis of primary CC, stage IIA (pathology T-stage 3, N0), microsatellite-stable primary tumors, and no adjuvant chemotherapy. Standard clinical and histopathological variables (TNM, grade, etc.) were retrieved for all patients. The “early relapse” group was defined as those patients experiencing a relapse within 5 years from the date of diagnosis, while the “no relapse” group consisted of patients who did not experience a relapse for at least 6 years. The relapse was defined as any disease recurrence or disease-related death except for any second primary cancers. To the extent possible, the two groups were further matched in terms of gender, age, and grade distribution. Failure of laboratory analyses (problematic sample preparation, low quality and/or quantity of isolated RNA, and low quality of expression data) was a reason for excluding these samples from the study.

From each tumor block, two different regions were sampled in adjacent sections: one representing the bulk tumor and one only the invasion front (**Supplementary Figure 1**). Each sample was profiled independently.

### 2.2 Expression profiling

The RNA extraction was performed from formalin-fixed paraffin-embedded histopathological slides using AllPrep DNA/RNA Kits (Qiagen, Hilden, Germany) according to manufacturer’s instructions. The extracted RNA served as input for a GeneChip WT Pico Reagent Kit (Thermo Fisher Scientific, Waltham, MA, USA) for analysis of the transcriptome on whole-transcriptome arrays. Total RNA from HeLa cells provided in the kit was used as a positive control together with high-quality low-concentration RNA isolated from a serum as a low-input control. Clariom D Array for human samples (Thermo Fisher Scientific, Waltham, MA, USA) was used for target hybridization to capture both coding and multiple forms of non-coding RNA. Finally, the arrays were scanned using Affymetrix GeneChip Scanner 3000 7 G (Thermo Fisher Scientific, Waltham, MA, USA). All the samples complied with the quality control requirements, and none of the samples were excluded from the analysis.

### 2.3 Bioinformatics analyses

All resulting CEL files were processed using Bioconductor (17) (v.3.15) packages oligo (18) (v.1.60), affycoretools (v1.68), and, for Clariom D chip annotation, pd.clariom.d.human (v.3.14). For the quality control, we used AffyPLM (v.147) and imposed a maximal median Normalized Unscaled Standard Error (NUSE) of 1.12. All chips passing the quality control steps were normalized together using RMA (oligo) with core-probeset summarization. Further, the array data were summarized at gene level by selecting the most variable probeset per unique EntrezID, and entries corresponding to missing HUGO symbols, speculative transcripts, microRNA, and short non-coding RNA were discarded resulting in a reduced list of 28,663 unique genes.

For the identification of differentially expressed genes, we used linear models (limma package v.3.52.2) with a cut-off for false discovery rate (FDR) of 0.1. The pathways were scored in terms of enrichment in specific signatures using gene set enrichment analysis (GSEA) (19) as implemented in fgsea package (v.1.22.0). MSigDB (hallmark gene sets collection “H” v.7.4.1) (20) was used as the main source for gene sets and pathways. The gene expression classifiers were based on ElasticNet model as implemented in the R package caret (v.6.0). All data analyses were performed in R 4.3 (R Development Core Team, 2022).

The development of the predictive models required the following two major steps: feature generation and classifier training. These two steps were embedded in an external leave-one-out loop for estimating the performance. The main performance parameter of the model was the area under the receiver operating curve (AUC) with sensitivity and specificity also estimated and reported. For the feature generation step, we first selected the most predictive (in terms of AUC) and stable genes and grouped them into modules according to gene signatures from MSigDB (H-section). For estimating the stability of each gene, we generated *b* = 50 bootstraps of the current training set (at each iteration of the leave-one-out procedure) and recorded the AUC and direction of the association of the gene with the outcome. We defined the direction of a gene *g* as *d_g_* = +1 if the average expression of the gene in the “early relapse” group was higher than in the “no relapse” group; otherwise, *d_g_* = -1. The AUC for a gene was the average AUC from bootstrapping procedure, and the gene was considered stable if the direction of the association with the outcome was constant (over the *b* bootstraps). The gene modules were generated from MSigDB gene signatures by selecting the top five (in terms of AUC) subsets of *n_g_* genes from each signature. The value of a module was defined as n_g_^-1^ Σ *d_g_x^g^*, where *x_g_* is the expression of gene *g* in the module. By extension, the names of the gene modules were taken from the names of the corresponding signatures even though they no longer represented their de-/activation status. Then, an ElasticNet model was fitted on the top n_f_ gene modules. To minimize the chances of overfitting, the tested domain for *n_g_* and *n_f_* was limited to values 3, 4, and 5. No constraint was imposed on the number of times a gene could be selected in different modules (the signatures from MSigDB overlapped) nor on selecting only one module per gene signature. While this choice introduces potential redundancy in the model, it also improves its robustness.

To validate the modeling approach, we used two independent data sets (21) compatible with our experimental design (with the exception of unknown MSI status) publicly available from ArrayExpress under accession numbers E-MTAB-863 and E-MTAB-864, respectively. We further limited the set of genes to the intersection of the two platforms (Clariom D for our study and Affymetrix customized Almac array for the independent sets) resulting in 13,274 common symbols. Also, in the validation sets (denoted KEN1 and KEN2), we considered only the patients in our target group (pT3/pN0/pM0); the rest of the expression profiles were used for mitigating the differences between the two microarray platforms. The model built (and validated) on the restricted set of genes was considered as a baseline model. Additionally, as the two external data sets contained survival data as well, we estimated the probability of survival in the two predicted groups using the Kaplan–Meier estimator and tested for significant difference between the curves using the log-rank test.

The main analysis considered the full set of genes available on our platform (Clariom D) and concerned the two sampling regions as follows: bulk tumor and invasion front, respectively.

## 3 Results

In total, *n* = 39 patients were identified fitting the selection criteria [19 cases of early relapse (12 men) vs. 20 cases of no relapse (11 men)] resulting in 39 bulk tumor profiles. For the same patients, *n* = 35 [17 early relapse (11 men) and 18 no relapse (10 men)] good quality invasion front profiles were also generated. No statistically significant differences were found between groups regarding age, tumor location, or grade (**Table 1**).

**Table 1:** Basic patient population demographics for the training set.

	Early relapse (within 5 years)	No relapse (for at least 6 years)	p-Value	Test
**N**	19	20		
**Age [mean (SD)]**	69.5 (9.22)	68.9 (9.56)	0.849	Student’s t-test
Gender				
Female Men	7 12	9 11	0.747	Fisher’s exact test
Grade				
G2 G3	18 1	20 0	0.487	Fisher’s exact test
Tumor site				
Right (including transverse colon) Left	14 5	12 8	0.501	Fisher’s exact test

All patients were stage II/A, microsatellite stable.

### 3.1 Differentially expressed genes and pathways

**Figure 1:**
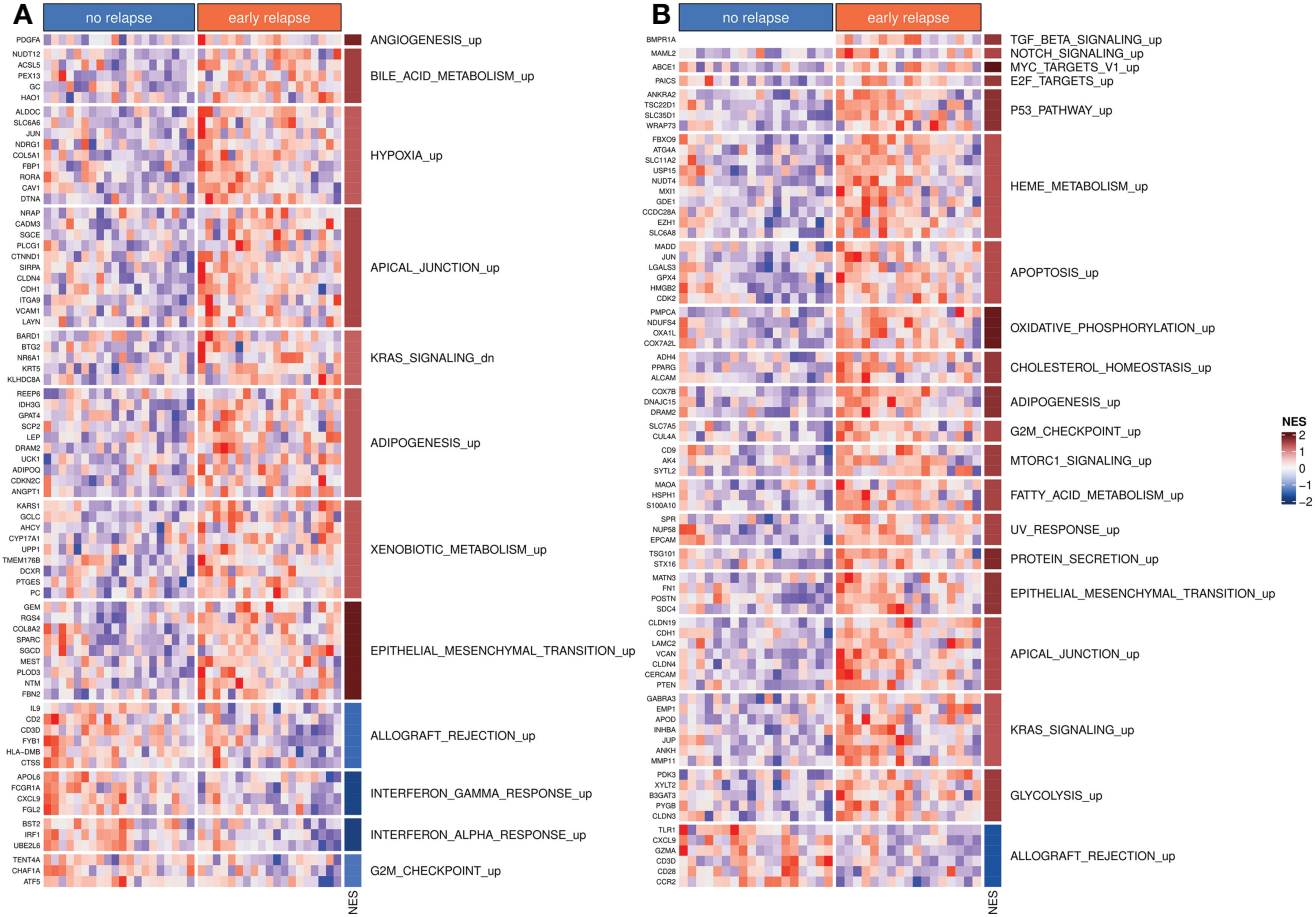
Differentially activated hallmark pathways. **(A)** Hallmark pathways and top differentially expressed genes from bulk tumor profiles. **(B)** Hallmark pathways and top differentially expressed genes from invasion front profiles. In both panels, *NES* indicates the normalized enrichment scores. The suffix “_up” or “_dn” indicates whether higher *NES* values correspond to set of gene sets that were activated (“_up”) or inhibited (“_dn”: down), respectively.

The differential expression analyses of both bulk tumor and invasion front samples revealed no genes with significantly different expressions between early and no relapse groups after adjusting for multiple testing. Nevertheless, 204 and 333 genes had a significant (un-adjusted) p-value (≤ 0.01) within the bulk tumor and invasion front samples, respectively. Using the *t*-statistics estimated by limma as input for ordering the whole set of genes for GSEA, we identified a number of pathways/gene sets differentially activated between the early relapse and no relapse groups (**Figure 1**). The full list of significant (un-adjusted p-value) genes (p-value ≤ 0.01) is given in **Supplementary Table 1** and the GSEA results in **Supplementary Table 2**.

### 3.2 Early relapse predictors

**Figure 2:**
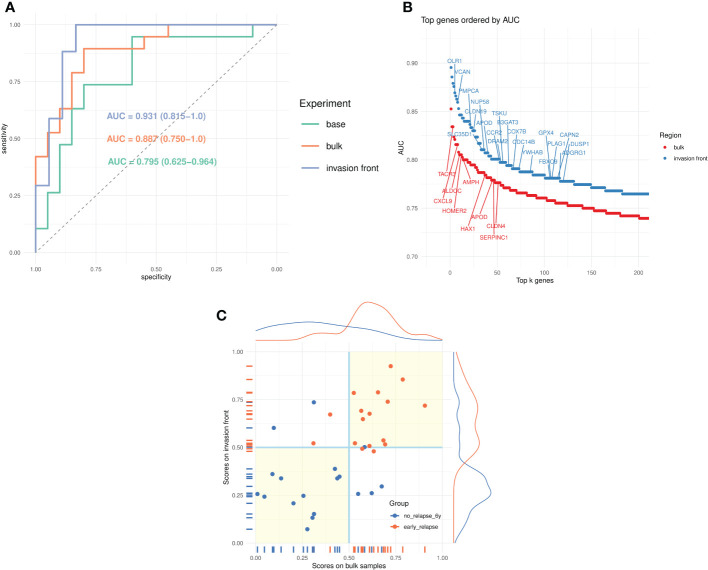
Prediction of early relapse. **(A)** Receiver operating characteristics (ROC) curves for the three models (baseline, bulk tumor, and invasion front) and the corresponding AUCs. **(B)** Univariate AUC, based on all samples, for top *k* = 200 genes from bulk tumor and invasion front expression profiles. The top genes (AUC > 0.775) from MSigDB hallmark signatures are marked. **(C)** Scatter plots of scores from bulk tumor and invasion front (35 samples) and their marginal distributions. The points are colored according to their true category, and the quadrants marked (light yellow background) indicate regions of agreement for the two classifiers.

To validate the approach, we developed a baseline predictor of early relapse cases using a restricted set of genes common to the two platforms (Clariom D and Almac) and based on bulk tumor profiles. The optimal model used *n_f_* = 5 gene modules each with *n_g_* = 4 genes (see **Table 2**). Its estimated leave-one-out performance was *AUC*_0_ = 0.795(95%*CI* = 0.625 – 0.964) (**Figure 2A**). The binary classification performance (for the default cut-off of 0.5) was sensitivity *Se* = 0.737(95%*CI* = 0.488 – 0.908) and specificity *Sp* = 0.8(95%*CI* = 0563 – 0.943). At the same time, the observed performance on the validation sets was *AUC_KEN_*_1_ = 0.731(95%*CI* = 0.636 – 0.827) and *AUC_KEN_*_2_ = 0.768(95%*CI* = 0.612 – 0.874) being superior to the one reported elsewhere (21) (**Supplementary Figure 2**). The Kaplan–Meier curves for predicted groups (“no relapse” and “early relapse”) were significantly different (*p* < 0.001) (**Supplementary Figure 3**).

**Table 2:** Predictive models and their performance.

Model	Modules and coefficients	Module coefficient	Genes in modules	Leave-one-out performance estimates (with 95% confidence intervals)
**Baseline model**	INTERFERON_GAMMA_RESPONSE_up1 INTERFERON_GAMMA_RESPONSE_up2 INTERFERON_GAMMA_RESPONSE_up3 TNFA_SIGNALING_VIA_NFKB_up1 TNFA_SIGNALING_VIA_NFKB_up2	1.0545 −0.7575 2.0305 1.8225 −0.9185	LATS2 - IRF1 - TRIM14 - APOL6 LATS2 - CXCL9 - TRIM14 - APOL6 LATS2 - IRF1 - TRIM14 - CXCL9 DUSP1 + LAMB3 - IRF1 - SLC2A6 DUSP1 + JUN - IRF1 - SLC2A6	AUC = 0.795 (0.625–0.964) Se = 0.737 (0.488–0.908) Sp = 0.8 (0.563–0.943)
**Bulk tumor model**	INFLAMMATORY_RESPONSE_up IL6_JAK_STAT3_SIGNALING_up APICAL_JUNCTION_up	1.1161 −0.1483 0.6747	EBI3 + KCNMB3 + TLR2 - IRF1 - TACR3 EBI3 + HAX1 + TLR2 - IRF1 - CXCL9 CLDN4 + LAYN + ITGA9 + NRAP + CADM3	AUC = 0.887 (0.75–1.0) Se = 0.895 (0.669–0.987) Sp = 0.75 (0.509–0.913)
**Invasion front model**	APICAL_JUNCTION_up KRAS_SIGNALING_up HEME_METABOLISM_up	0.1652 0.1527 0.0915	VCAN + CLDN19 + PTEN + CDH1 GABRA3 + APOD + JUP - TMEM100 EZH1 + CCDC28A + FBXO9 + SLC6A8	AUC = 0.931 (0.815–1.0) Se = 0.882 (0.636–0.985) Sp = 0.833 (0.586–0.964)

For the genes in the modules, a positive sign (explicit or implicit) indicates its higher expression in the “early relapse” group, while the negative sign indicates the reverse situation.

With the modeling approach validated, we studied the predictive power of the profiles derived from bulk tumor and invasion front regions. First, we compared the univariate (per-gene) AUCs for bulk and invasion front profiles (**Figure 2B**, **Supplementary Table 3**) estimated using all samples. It was apparent that the invasion front expression profiles were more predictive with the top ranking genes having consistently higher univariate AUC (2%–5%). Also, there were almost twice as many genes from the invasion front with AUC > 0.7 than from bulk tumor profiles (**Supplementary Table 3**).

The predictors built from the bulk and invasion front profiles confirmed this tendency (**Figure 2A**): the leave-one-out estimated performance for invasion front was *AUC_i_* = 0.931(95%*CI* = 0.815 – 1.0)(*Se* = 0.882,*Sp* = 0.833), superior to the bulk tumor performance: *AUC_b_* = 0.887(95%*CI* = 0.750 – 1.0)(*Se* = 0.895,*Sp* = 0.75). The two models are given in **Table 2** and further gene annotations in **Supplementary Table 4**.

### 3.3 Combining predictors

We also compared the scores (posterior probabilities from ElasticNet models) produced by the two models (**Figure 2C**). The correlations (Pearson correlation: 0.564, Spearman correlation: 0.582) between the scores were modest, as was Cohen’s kappa coefficient (κ = 0.484 between the class assignments based on these scores). This indicated a certain degree of complementarity between the two models, and we speculatively created an average score (from leave-one-out scores of matched tumor bulk and invasion front samples) and used it for predicting the groups. The new score indeed improved on all previous predictions—*AUC* = 0.977(95%*CI* = 0.907 – 1.0),*Se* = 0.941,*Sp* = 0.889).

## 4 Discussion

The intermediate-risk group of patients with stage II colon cancer is heterogeneous in terms of survival experience: while most of the patients fare well without any adjuvant chemotherapy, others relapse much sooner. Reliably identifying the patients at risk for early relapse is, therefore, fundamental.

Our pilot study addressed two problems: First, developing a gene-based predictor for the stage IIA colon cancer patients who, despite being considered as low risk of relapse by current guidelines, are relapsing within 5 years. The second problem addressed aimed at investigating whether the invasion front is more predictive for the early relapse. Benefitting from a matched data set on which both bulk tumor and invasion front were profiled, we developed two predictive models. In our data, the invasion front model proved to be significantly superior to the bulk tumor model. This suggests that the dynamic changes happening on the contact border between the tumor and the normal tissue of the host may bear more information about the invasiveness potential of the tumor.

The targeted patient population appears to be rather homogeneous from the perspective of transcriptomics, with no gene significantly differentially expressed between “no relapse” and “early relapse” groups, after adjustment for multiple hypotheses testing. Nevertheless, several genes reached statistical significance when considered individually with more genes in the case of invasion front samples. Using the results from the differential expression analysis as input for gene set enrichment analyses, several significantly deregulated pathways/gene sets were identified. Some of them were common between bulk tumor and invasion front samples, most notably the epithelial-to-mesenchymal transition pathway, which was strongly up regulated in early relapse cases. Interestingly, the KRAS activation appeared in contrasting instances between the following two types of samples: in bulk tumors, the KRAS-down gene set was activated in the “early relapse” group, while in invasion front samples, the KRAS-up gene set was activated in the same group of patients, indicating a differential activation of KRAS between bulk tumor and invasion front regions within early CC.

The first predictor for early relapse established a baseline model and performance and validated the modeling approach. However, it was limited in the number of genes covered, as the two independent validation sets originated from an older microarray platform. Nevertheless, we were able to construct and validate a relatively strong classifier from bulk tumor profiles. The validation sets (21) were not selected for MSS, as this was not reported, but the baseline model performed close to the estimated performance. While the baseline classifier relied on five gene modules, the features selected by the algorithm referred to only two of the following MSigDB’s pathways: interferon-gamma (INF-γ) and tumor necrosis factor-alpha (TNF-α) via nuclear factor-κβ, related to antitumor immunity and inflammatory processes, respectively. More interestingly, one gene—*IRF1* (interferon regulatory factor 1)—was common to both pathways (and to both bulk tumor models) and selected in four out of five modules being downregulated in the early relapse group. Upregulation of this gene was shown to be related to better survival and tumor radiosensitivity (22). We also note that the model could be further simplified to a model with only two modules (INF-γ and TNF-α) each of five genes; however, this combination was not foreseen when training the models (we imposed *n_f_* = 3,4,V 5).

The same modeling approach was applied on tumor bulk and invasion front profiles considering all the genes present on our platform (still limited to the hallmark pathways of MSigDB). This led to the development of two models of which the invasion front signature had the best performance while both being superior to the baseline model. As the models were derived from tumor samples originating from the same patients, comparing the two allowed us to gain more insights into the predictive power of the invasion front. We first investigated the predictive utility (in terms of AUC) of each gene and found more genes from the invasion front having higher AUCs than from bulk tumors (see also **Supplementary Table 3**). While these results hinted toward more prognostic value of the invasion front signatures, it was the multivariable models (ElasticNets) that showed this being true in practice. Both models comprised of three gene modules with apical junction being a common term. However, the genes selected in the two “apical junction” modules were different with those from the invasion front pointing also toward EMT (*VCAN*) and estrogen receptor (*CDH1*). Also, we note the KRAS-related module present in the invasion front signature, which, corroborated with the results of GSEA (**Figure 1**; **Supplementary Table 2**), points toward a stronger KRAS pathway activation in early relapse patients. While specific mutations of the *KRAS* oncogene were shown to be predictive for overall survival in some studies (23, 24), they appeared not to be predictive for relapse-free survival (25). A more detailed annotation of all genes, with further references, is given in **Supplementary Table 4**. We also noted that the proposed marker gene for invasion front (15), *ZEB2*, was prognostic in our data as well, but with lower performance [AUC_ZEB2 = _0.716 (0.521–0.910); **Supplementary Table 3**].

Our pilot study has some limitations as well: the invasion front signature could not be validated on external independent data because no similar data collections exist. We make our data publicly available to begin filling this gap. Second, the sample size did not allow for more analyses. For example, the observation that combining invasion front and bulk tumor signatures into a stronger predictor was made *post hoc*, and it would require another data set for its statistical assessment.

Another aspect pertains to the definition/delineation of the invasion front. We expect a relatively significant inter-observer variability. Thus, for the future results to be validated independently, a consensus must be reached between pathologists to stabilize the sampling regions.

In conclusion, our study proposes a novel invasion front-derived gene signature for predicting high-risk patients within the stage IIA colon cancer group. Its combination with bulk tumor signature further improved the prediction suggesting that a combined, dual sampling of core and border of the tumor may lead to a practical and precise predictor.

## Data availability statement

The datasets presented in this study can be found in online repositories. The names of the repository/repositories and accession number(s) can be found below: https://www.ebi.ac.uk/arrayexpress/, E-MTAB-13695.

## Ethics statement

The studies involving humans were approved by Research Ethics committee of Masaryk University. The studies were conducted in accordance with the local legislation and institutional requirements. The participants provided their written informed consent to participate in this study.

## Author contributions

EB: Conceptualization, Formal analysis, Investigation, Methodology, Supervision, Writing – original draft. MČ: Methodology, Writing – original draft. TI: Investigation, Methodology, Writing – original draft. TM: Data curation, Writing – original draft. MB: Data curation, Writing – original draft. LP: Data curation, Writing – original draft. OS: Conceptualization, Methodology, Writing – original draft. BB: Data curation, Funding acquisition, Investigation, Project administration, Writing – original draft. VP: Conceptualization, Formal analysis, Funding acquisition, Investigation, Methodology, Project administration, Supervision, Writing – original draft.
