# Supplementary material for: An invasion front gene expression signature for higher-risk patient selection in stage IIA MSS colon cancer
Source: Front Oncol. 2024 Apr 19;14:1367231. doi: 10.3389/fonc.2024.1367231 (PMC11066151; doi:10.3389/fonc.2024.1367231)
Supplement: Supplementary file 1 [file DataSheet_1.docx]

Supplementary Material

# Supplementary Figure 1


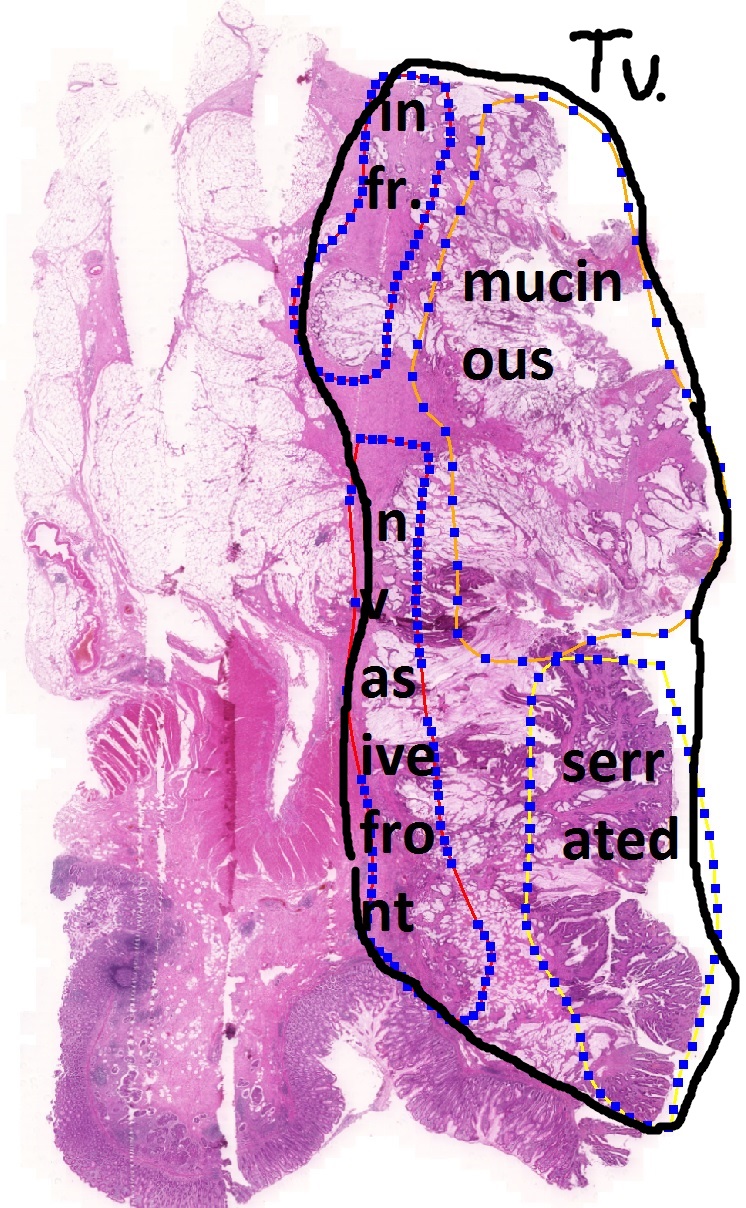
**Supplementary Figure 1.** Examples of tissue regions macrodissected for RNA profiling. The images show the regions marked by an expert pathologist on the pathological slide. The bulk tumor (marked “Tu” and solid black line) and invasion front (“inv. front” or similar annotation) were extracted from serial sections of the tumor.


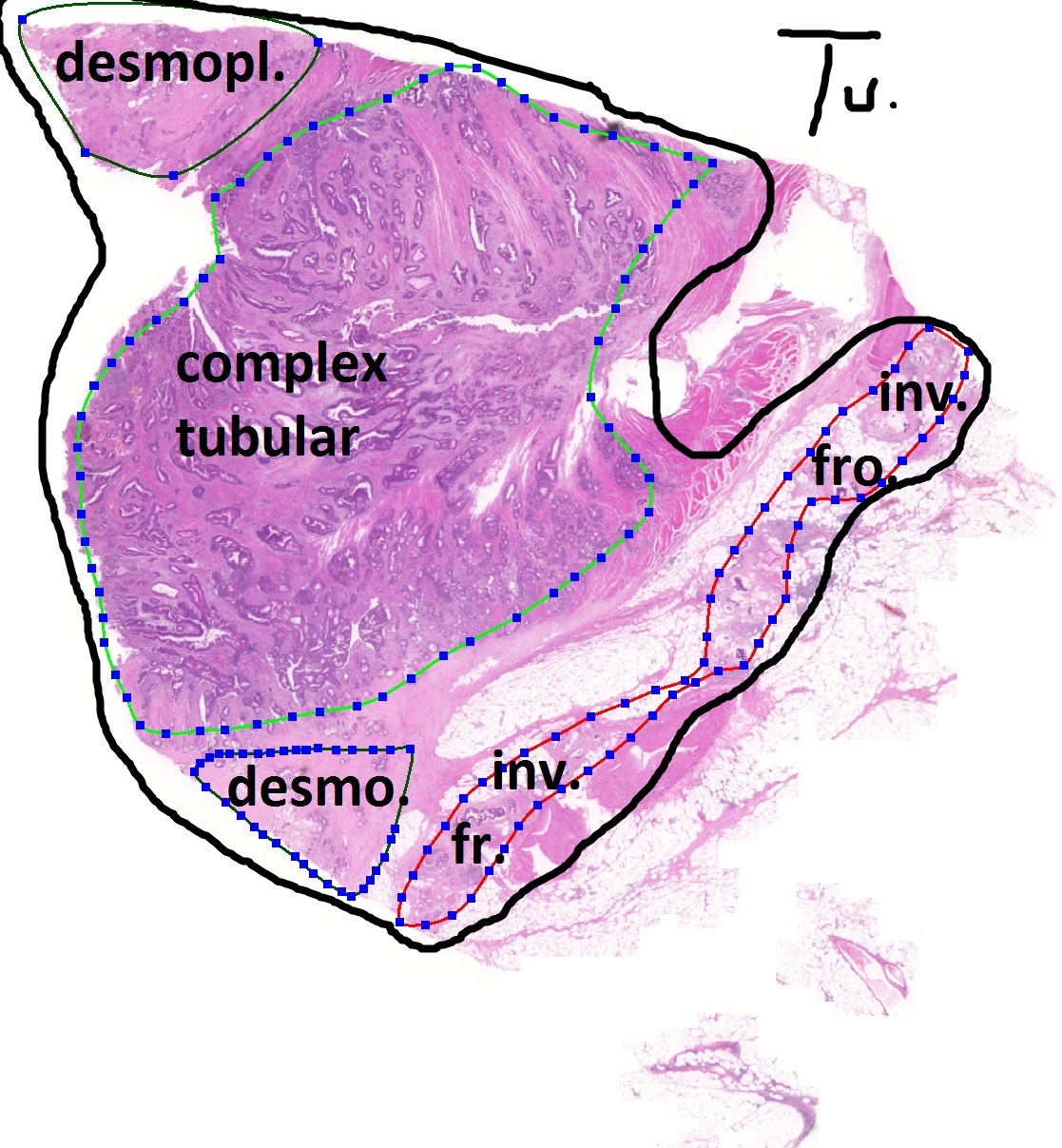

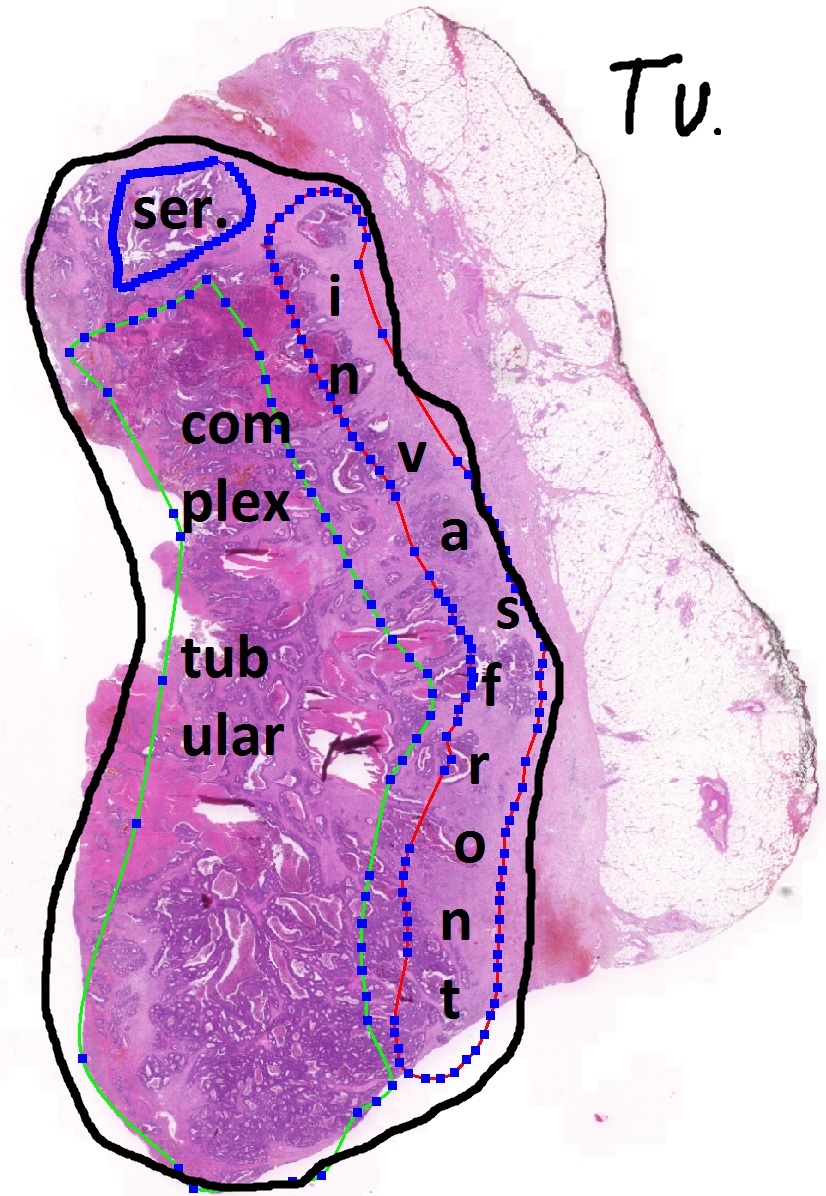

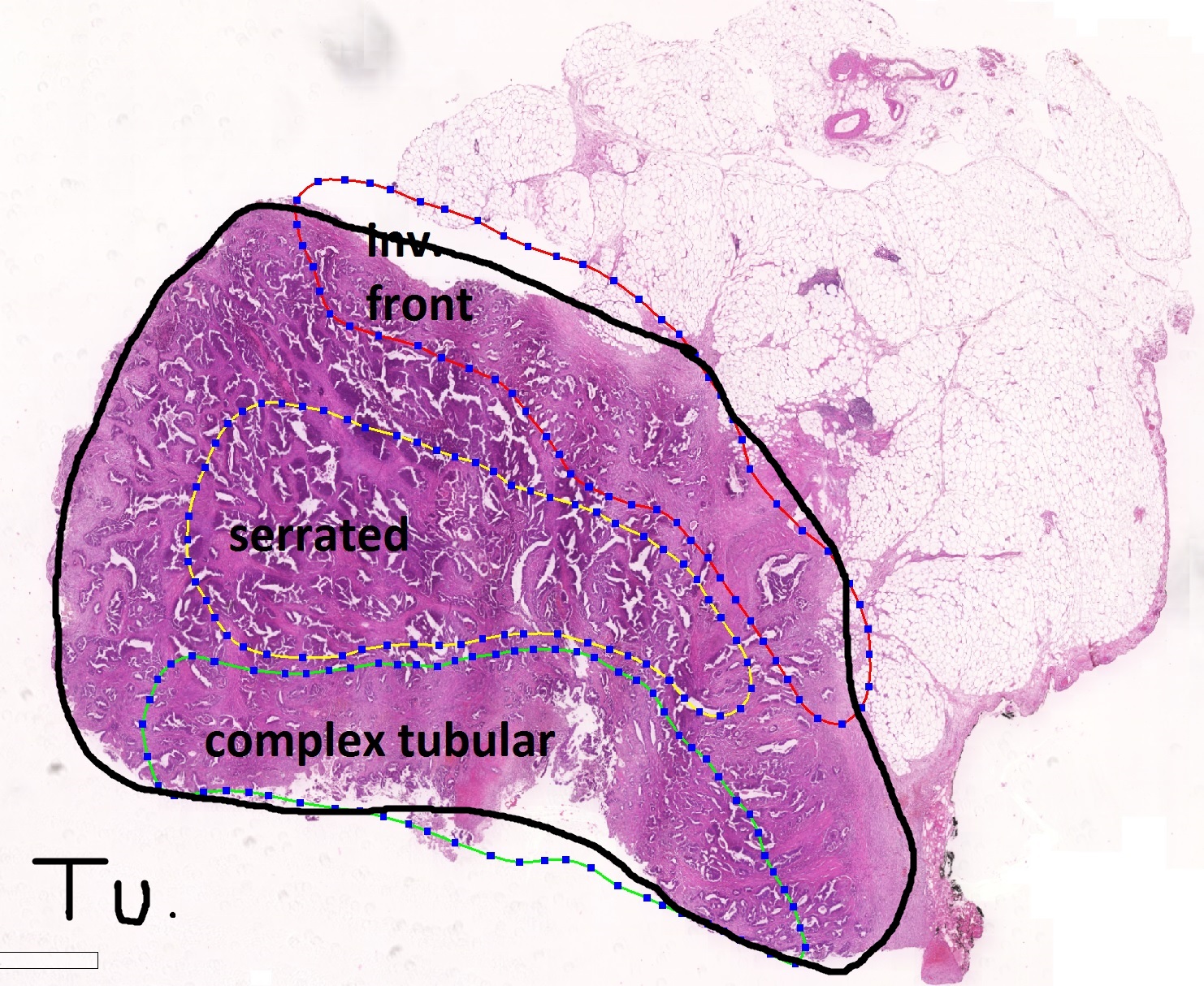


# Supplementary Figure 2


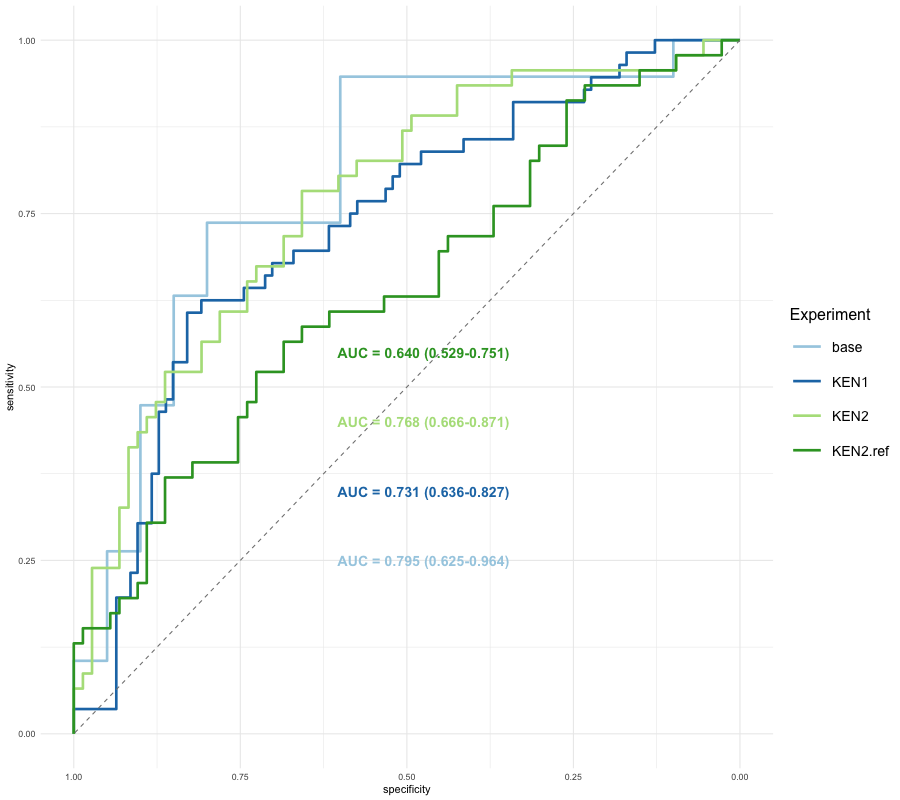


Supplementary Figure 2. Receiver operating characteristics curves (ROCs) for training and validation data. The training ROC ("base" line) is obtained from leave-one-out predictions. The validation ROCs are predictions made from the fitted model on all training data ("KEN1" and “KEN2” lines). The “KEN2.ref” curve indicates a reference ROC obtained from the scores predicted by the model in (1) and available in the data set E-MTAB-864.

# Supplementary Figure 3


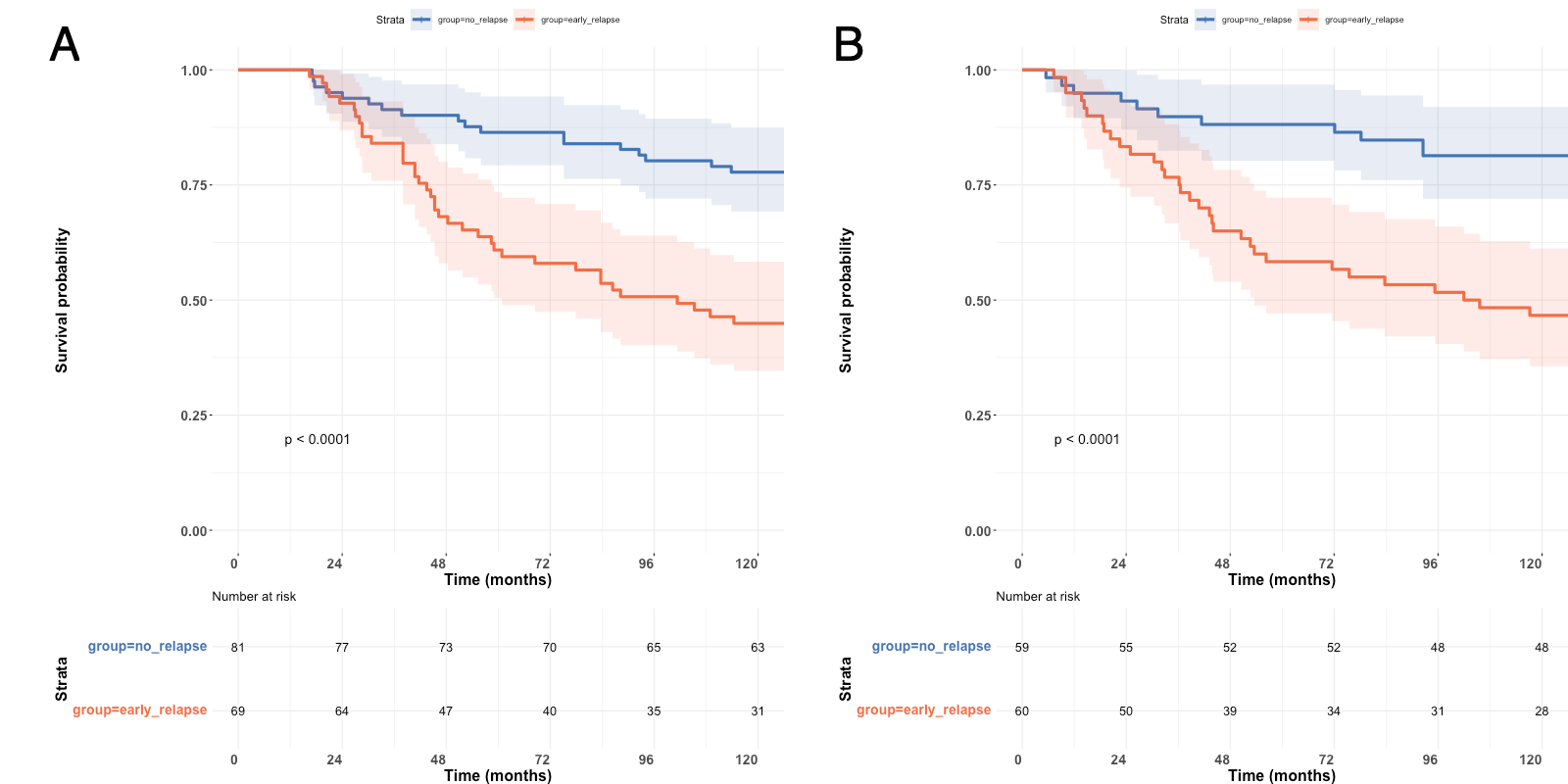


Supplementary Figure 3. Kaplan-Meier curves for the two validation sets - (A) KEN1 and (B) KEN2. The cut-off point value was the default value (0.5).

1. Kennedy RD, Bylesjo M, Kerr P, Davison T, Black JM, Kay EW, Holt RJ, Proutski V, Ahdesmaki M, Farztdinov V, et al. Development and Independent Validation of a Prognostic Assay for Stage II Colon Cancer Using Formalin-Fixed Paraffin-Embedded Tissue. *JCO* (2011) 29:4620–4626
